# Global trends and hotspots in blood glucose management for elderly patients with diabetes: A bibliometric analysis (2000–2024)

**DOI:** 10.1097/MD.0000000000043919

**Published:** 2025-08-15

**Authors:** Li Zhou, Lubi He

**Affiliations:** a Department of Endocrinology, Chengdu Eighth People’s Hospital (Geriatric Hospital of Chengdu Medical College), Chengdu, China; b Department of Nephrology, Affiliated Hospital of Sichuan Nursing Vocational College (Sichuan Province Third People’s Hospital), Chengdu, China.

**Keywords:** bibliometric analysis, blood glucose management, collaborative networks, diabetes, research trends

## Abstract

**Background:**

Effective management of blood glucose levels in elderly patients with diabetes is crucial in healthcare and has seen significant advancements. However, there is a lack of bibliometric analyses in this field. This study aimed to provide a comprehensive overview through bibliometric analysis.

**Methods:**

A bibliometric analysis was conducted using the Web of Science Core Collection to identify research trends in blood glucose management for elderly patients with diabetes from 2000 to 2024. The analysis utilized VOSviewer version 1.6.20, CiteSpace version 6.3.R1, and R version 4.3.3 to visualize collaboration networks and emerging research trends.

**Results:**

A total of 11,826 publications were analyzed, sourced from 134 countries and 228 institutions between 2000 and 2024. There has been a consistent increase in publications, particularly from 2020 to 2022. The United States was the leading contributor, with 2436 publications, while Harvard University had the highest output at 829 publications. Among the journals, *Diabetes Care* was the most impactful based on its high Hirsch index. Influential authors included Beck Roy W and Khunti Kamlesh. Keyword analysis identified “continuous glucose monitoring,” “weight,” “progression,” “young adults,” “classification,” and “time in range” as primary research themes since 2017.

**Conclusion:**

This bibliometric analysis provides a detailed overview of blood glucose management in elderly diabetic patients. Key focus areas such as “continuous glucose monitoring,” “time in range,” and “weight” have gained significant attention. Future research should further explore these themes, especially the role of continuous glucose monitoring and the concept of time in range in clinical practice.

## 1. Introduction

Diabetes mellitus poses a significant challenge to global healthcare, particularly as it pertains to the elderly population.^[1–3]^ This demographic is disproportionately affected due to the interplay of age-related physiological changes, comorbidities, and the complexity of managing blood glucose levels.^[4–6]^ Moreover, the number of elderly diabetic patients may grow to 253 million in 2045.^[7]^ The diabetes mellitus is also risk factors of cognitive decline and dementia,^[8]^ diabetic foot ulcers,^[9]^ retinopathy and ocular surface damage,^[10,11]^ and cardiovascular disease,^[12]^ contributing huge burden and poor quality of lives. Thus, the prevalence of diabetes in the elderly is a growing concern, underscoring the necessity for tailored research and interventions that address the unique needs of this group.^[13–15]^

Effective blood glucose management is pivotal in mitigating diabetes-related complications and enhancing the quality of life for elderly diabetic patients.^[16–19]^ The traditional drug therapy to diabetes care, often generalized for all age groups, may not be optimally effective or safe for the elderly, which can trigger serious cardiovascular events in older people.^[20]^ This underscores the importance of research that is specifically geared towards the geriatric considerations in diabetes management. Although several therapy options are available for the treatment of diabetes in older people,^[21]^ data are limited in this age group. There is less information available on pharmacological agents for older people than there is for younger individuals, and research studies are less likely to include older people with multiple comorbidities.^[20]^ In light of these contextual factors, this research delves comprehensively into the clinical practice of blood glucose management in elder patients.

The vast quantity of research-related literature currently being produced presents a challenge for traditional literature analysis in obtaining comprehensive and pertinent information. Bibliometrics has emerged as an interdisciplinary domain that harnesses mathematical and statistical techniques for the quantitative and qualitative dissection of scholarly literature.^[22]^ This methodology facilitates a multifaceted examination of publications, offering insights into the publication landscape. It encompasses the participation of countries, institutions, journals, and authors, and is instrumental in identifying seminal works and pivotal discoveries.^[23]^ The present study leverages bibliometric analysis to delineate the global research trends in blood glucose management among elderly diabetic patients. The study systematically examines the scholarly output to identify influential publications, key research hubs, and dominant thematic areas. By providing an overview of the research landscape, this study aimed to elucidate the logical progression of research themes and to forecast potential future directions in the field.

## 2. Materials and methods

### 2.1. Search strategies and data collection

A comprehensive literature search was conducted on the Web of Science Core Collection, focusing on the topic of blood glucose management in elderly patients with diabetes, covering the period from 2000 to 2024. The search strategy employed a detailed formula as follow #1 (((((((TS = (elderly)) OR TS = (aged)) OR TS = (old age)) OR TS = (senile)) OR TS=(“elderly patients”)) OR TS = (elder)) OR TS = (older)) OR TS = (aging). #2 (((((((TS=(“diabetes mellitus”)) OR TS = (diabetes)) OR TS = (diabetic)) OR TS=(“diabetic mellitus”)) OR TS=(“experimental diabetic”)) OR TS=(“type 2 diabetes mellitus”)) OR TS = (diabete)) OR TS = (diabetics). #3 (((((TS=(“blood glucose”)) OR TS=(“blood sugar”)) OR TS = (glucose)) OR TS=(“plasma glucose”)) OR TS=(“serum glucose”)) OR TS = (glycemia). #4 (((((((TS = (management)) OR TS = (administration)) OR TS=(“is managed”)) OR TS=(“s management”)) OR TS = (manage)) OR TS = (administrant)) OR TS = (regulation)) OR TS = (management) #5 #1 AND #2 AND #3 AND #4. The search was restricted to publications in English to ensure uniformity in language. Only articles were selected for inclusion. To minimize the impact of database updates on the search results, the literature retrieval was performed on a single day, August 9, 2024. All relevant data were extracted in text format, including the number of publications and citations, titles, author information, institutional affiliations, geographical regions, keywords, and journal names. This information was meticulously collected to facilitate a thorough bibliometric analysis, which would provide valuable insights into the research landscape of blood glucose management in elderly patients with diabetes.

### 2.2. Statistical analysis and visualization

A meticulous bibliometric analysis was performed, leveraging a comprehensive set of analytical and visualization tools. R 4.3.3 was instrumental in generating descriptive analysis results, an exercise synonymous with performance analysis in bibliometrics.^[24]^ This analysis encompassed a broad range of indicators, such as the total number of publications, average citations per publication, and data pertaining to countries, institutions, journals, and authors. The Hirsch index, which quantifies the number of papers with a citation count exceeding their position in the citation sequence,^[25]^ and the impact factor were extracted from the most recent Journal Citation Reports. Subsequently, VOSviewer 1.6.20, renowned for its ability to map collaborations, including co-authorships, citations, and co-citations, was employed to visualize and explore the intricate academic relationships. Furthermore, to delineate emerging trends and research focal points, a co-occurrence analysis of keywords was conducted using VOSviewer, complemented by keyword detection facilitated by CiteSpace 6.3.R1. CiteSpace 6.3.R1 was utilized for the keyword co-occurrence analysis, which spanned publications from January 1979 to June 2024.

## 3. Results

### 3.1. Overview of publications

**Figure 1.**
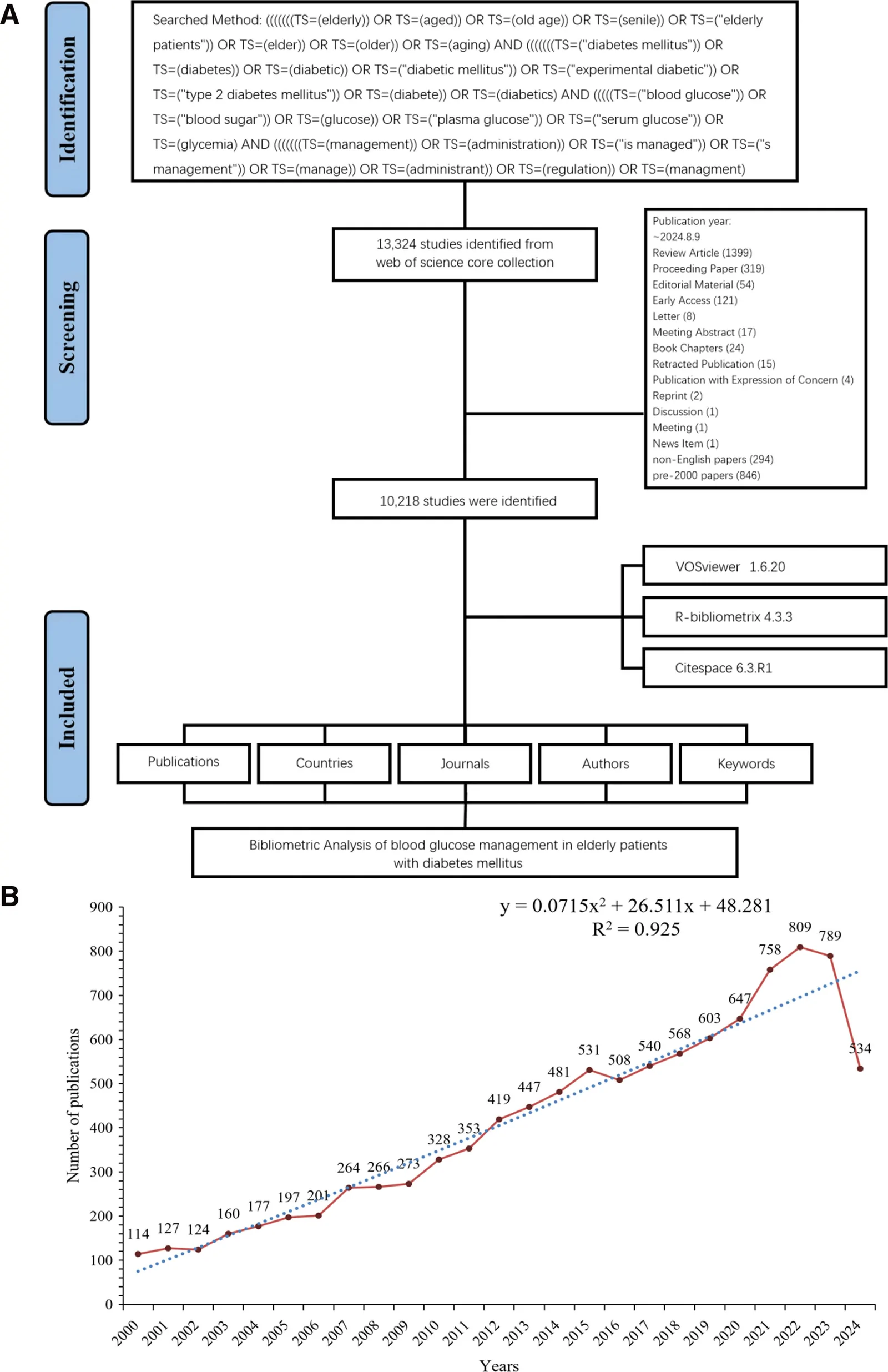
(A) Flowchart of the literature screening process. (B) Annual number of publications and trend from 2000 to 2024.

The details pertaining to the literature extraction process were shown in Figure 1A. From the search strategy, this study examined 13,324 publications concerning this field from 2000 to 2024. Ultimately, this investigation showed that 65,079 authors from 38,727 institutions across 1041 countries/regions contributed to the production of 11,826 manuscripts in this study. These works were published in 1819 journals, citing 247,655 references. An overall increasing trend in publications has been steadily rising since 2000 (Fig. 1B), followed by a sharp rise in the number of publications from 2020 to 2022, suggesting a heightened interest in research pertaining to blood glucose management in elderly patients with diabetes during this period, peaking in 2022 with 809 publications. Moreover, a linear trend line of annual publications was created with equation *Y* = 0.0715*X*^2^ + 26.511*X* + 48.281, where *Y* represents the annual publications and *X* denotes the year. This model exhibits a coefficient of determination (*R*²) of 0.925.

### 3.2. Analysis of countries and regions

**Figure 2.**
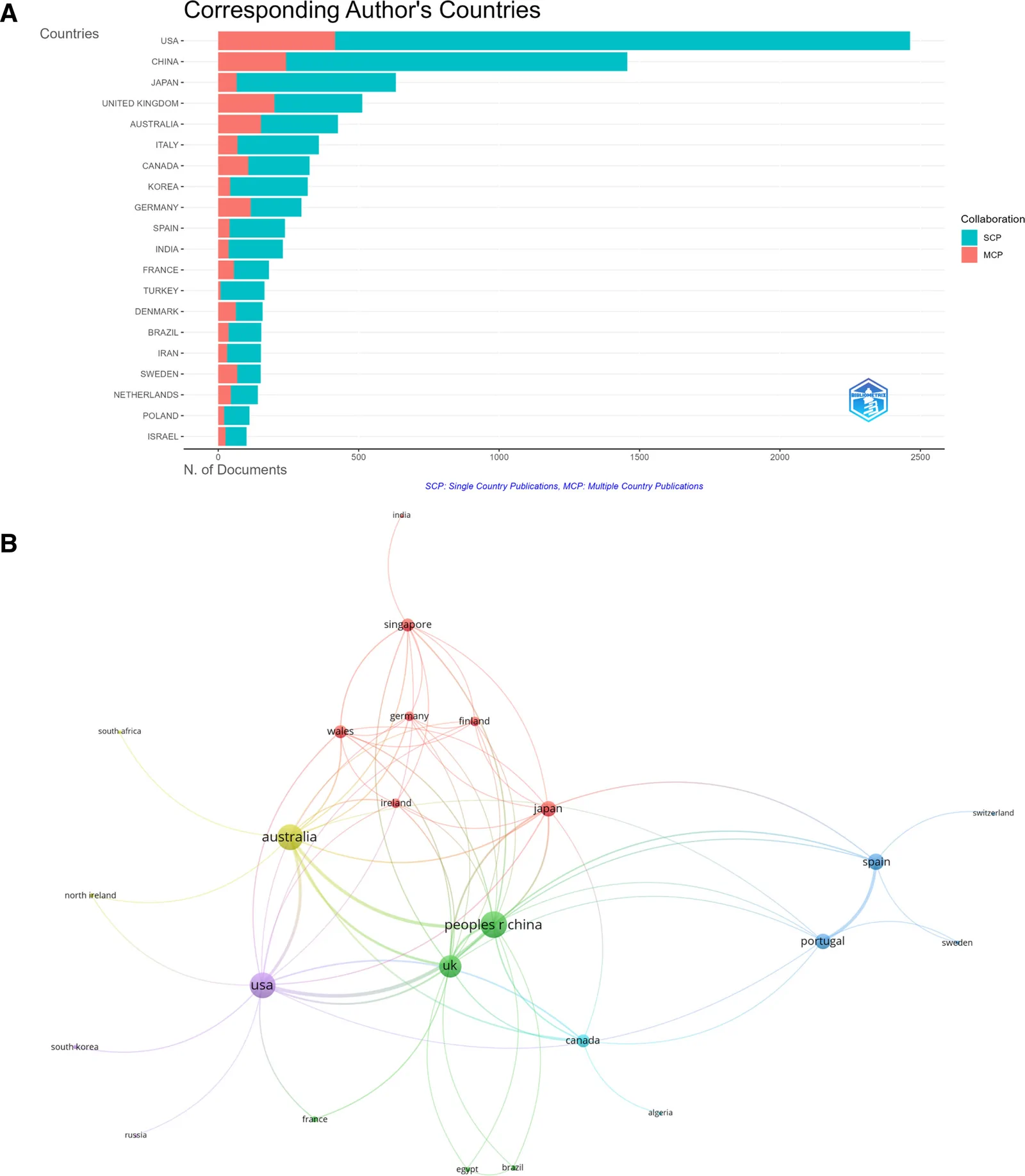
Global distribution in blood glucose management in elderly patients with diabetes. (A) Distribution of corresponding authors’ publications by country. (B) Visualization Map depicting the collaboration among different countries.

A total of 1041 countries/regions published articles on the topic at hand. The top 20 productive countries generated articles, accounting for 72.5% of the papers worldwide (Fig. 2A). Among the top 20 productive countries, the USA was the most productive country with the highest articles (2463), followed by China (1457) and Japan (633). For country collaboration, the USA ranks first and leads the way in multiple country publications (n = 416), followed by China (242) and the United Kingdom (n = 200). Besides, the USA occupied the leading role of average citations (52.5), followed by the United Kingdom (51.9) and the Sweden (51.6) (Table S1, Supplemental Digital Content, https://links.lww.com/MD/P687). Among the 110 countries involved in international collaborations with a minimum of 1 article, China has the highest number of collaborations with other countries (64), followed by Australia (58) and the USA (57) (Fig. 2B).

### 3.3. Analysis of institutions

**Figure 3.**
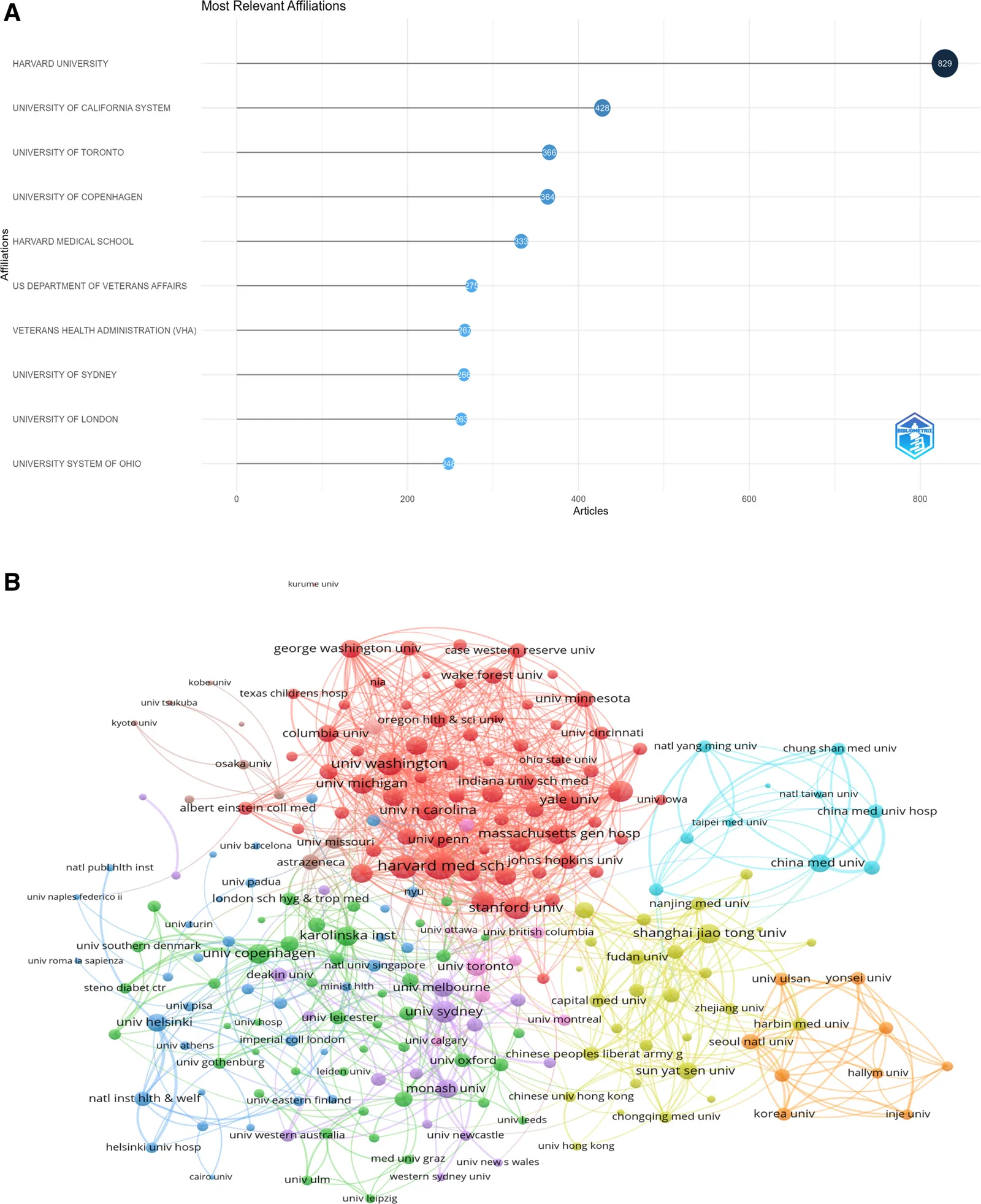
Institutional contributions and collaborations in blood glucose management in elderly patients with diabetes. (A) Top 10 institutions by article count and rank. The circle size shows the article count, with darker shades indicating higher ranks. (B) Visualization map depicting the collaboration among different institutions. Nodes represent institutions, with size indicating publication count. Links represent co-authorships, with thickness showing collaboration strength.

A total of 38,727 institutions were involved in research on this field. The top 10 institutions are displayed in Figure 3A. Harvard University has the highest number of publications (829), followed by University of California System (428) and University of Toronto (366). Notably, 6 out of the top 10 institutions are affiliated with the USA (6/10), indicating the leading position of USA in this field.

Moreover, the collaboration analysis illustrated that international partnerships are a key feature of research in this area. Among the 228 institutions involved in international collaborations with a minimum of 22 articles, Harvard University has the highest number of collaborations with other countries (140), followed by Harvard Medical School (128) and University of Copenhagen (124), playing the central role that owned the closest cooperation with other institutions (Fig. 3B).

### 3.4. Analysis of journals

**Figure 4.**
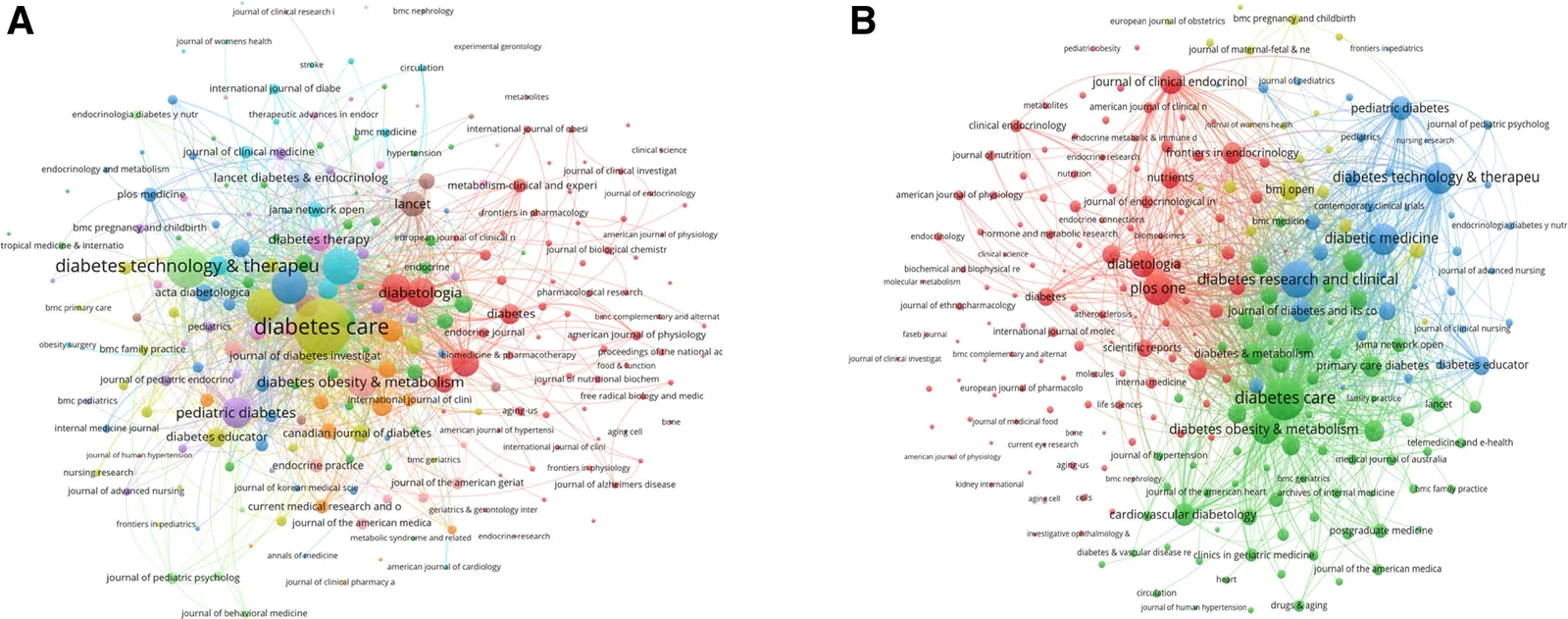
Visualization analysis of influential journals. (A) Co-occurrence Network of Journals. (B) Citation Coupling Network of Journals.

Upon analyzing the literature’s cited and citing journals, it was possible to determine the influential journals in the field. The bibliometric analysis of journals in this field revealed that all papers were published across 1819 academic journals. Notably, 20.8% of the studies were published in the top 20 journals based on their Hirsch index (Table S2, Supplemental Digital Content, https://links.lww.com/MD/P687). Among which, *Diabetes Care*, *Diabetes*, and *Diabetologia* stand out with the highest H_index (99, 61, and 61, respectively); *Lancet*, *Diabetes Care*, and *Metabolism-Clinical and Experimental* stand out with the highest journal’s impact (impact factor, Journal Citation Reports 2024 = 98.4, 14.8, and 10.8, respectively). The co-occurrence network and coupling network analysis included 242 journals with at least 8 occurrences in the dataset. The 3 key journals with the highest total link strength in co-occurrence networks were *Diabetes Care* (1388), *Diabetes Technology & Therapeutics* (695), and *New England Journal of Medicine* (521). In term of coupling network, the top 3 journals included were *Diabetes Care* (110,891), *Diabetes Research and Clinical Practice* (78,946), and *PLOS One* (59,219) (Fig. 4A, B).

### 3.5. Analysis of authors

**Figure 5.**
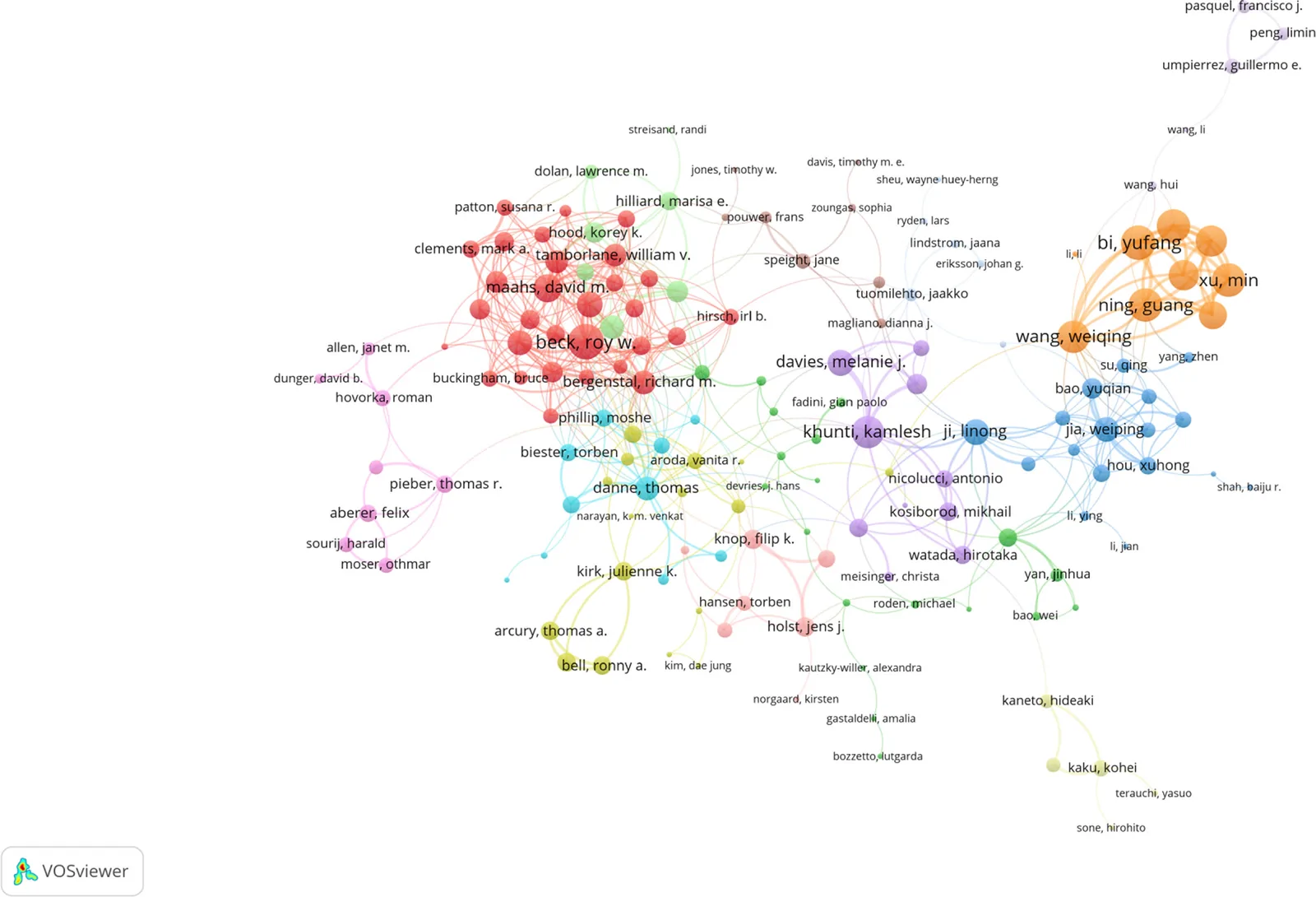
Visualization map depicting the collaboration among different authors. Nodes represent authors, with size indicating publication count. Links represent co-authorships, with thickness showing collaboration strength.

A total of 65,079 authors contributed to this field’s publications from 2000 to 2024. The top 20 high-impact authors based on h_index. The author Beck Roy W and Khunti Kamlesh ranked first with H_index of 22, followed by Danne Thomas and Davies Melanie J (H_index = 16) (Table S3, Supplemental Digital Content, https://links.lww.com/MD/P687). Furthermore, Beck Roy W and Khunti Kamlesh emerge as the high-impact authors, with their work being referenced 6253 times and 1812 times, which further emphasizes their pivotal roles in advancing the field. The collaborative network among 50 authors incorporated a minimum threshold of 14 articles per author (Fig. 5). Evidently, Beck Roy W sustains a conspicuously high echelon of scientific impact within this research sphere. For highly cited papers, the article published on *New Engl J Med* by Knowler Wc in 2002 gained the highest total citations, followed by articles published on *Lancet* by Danaei G in 2011 and *Diabetes Care* by Toobert DJ in 2000. Moreover, the papers on *Lancet* by Ong KL had the highest normalized total citations, attracting high attention recently (Table S4, Supplemental Digital Content, https://links.lww.com/MD/P687).

### 3.6. Analysis of keywords and burst keywords

**Figure 6.**
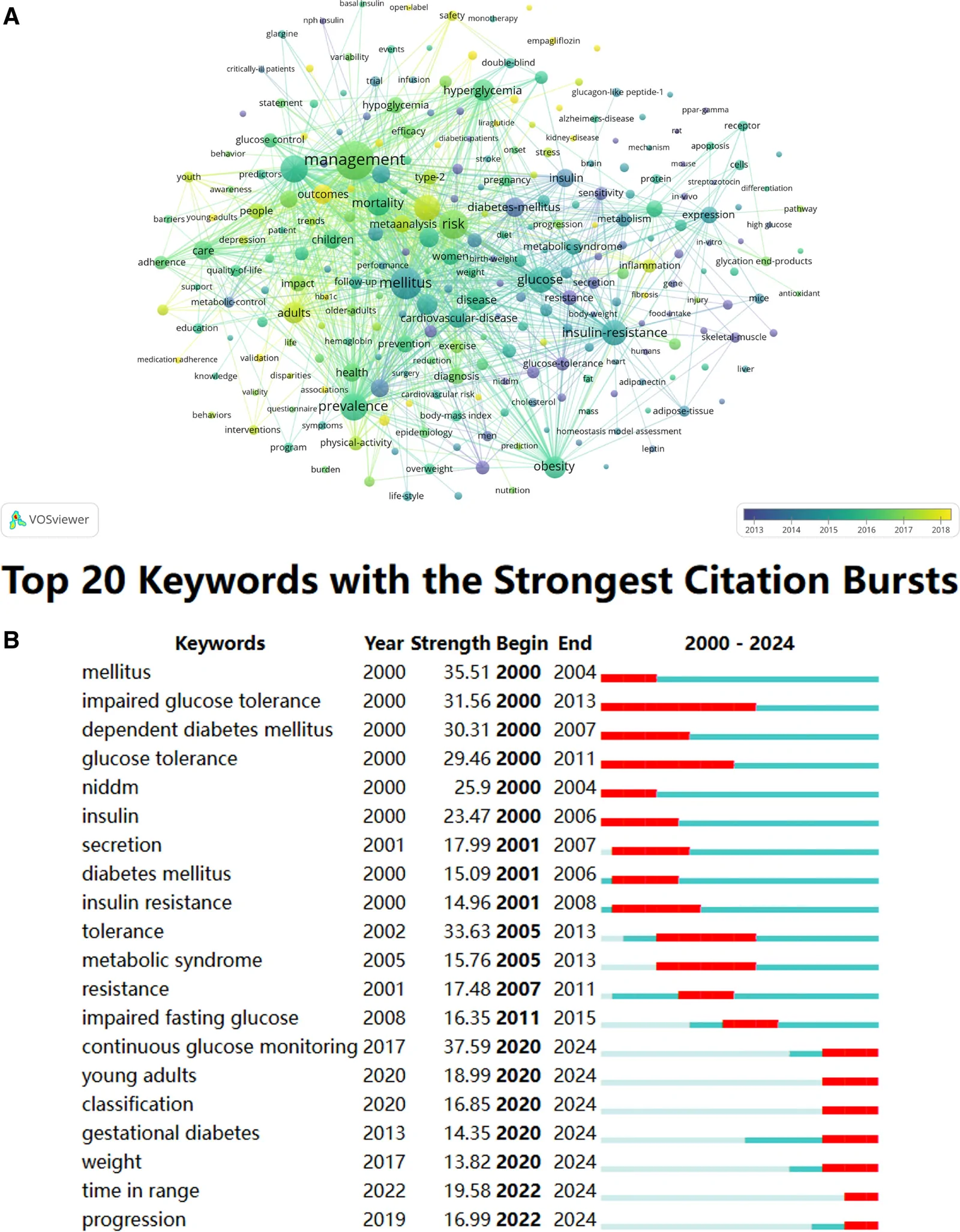
(A) Time-overlay visualization map of co-occurrence keywords. This network visualization displays the co-occurrence of keywords in selected literature. (B) Citation burst analysis of keywords in blood glucose management in elderly patients with diabetes. Top 20 keywords with the strongest citation bursts from 2000 to 2024.

Using VOSviewer, 261 keywords that appeared a minimum of 3 times across the articles were used to generate the time-overlay visualization map (Fig. 6A), ensuring a focus on the most prevalent and relevant terms in the field. The purple nodes indicate early hotspots and the yellow nodes indicate emerging hotspots and the main recent keywords are “management,” “outcomes,” “risk,” “adults,” highlighting the shift from diseases themselves to outcome and management in adult patients.

The burst analysis of keywords, conducted over a period spanning from 2000 to 2024, serves as a temporal lens through which the evolution of research focus can be observed (Fig. 6B). The intensity of the top 20 keywords with the most notable bursts ranged between 13.82 and 37.59, reflecting varying degrees of impact and attention within the research community. Prominently, the keyword “impaired glucose tolerance” exhibited the most pronounced burst, suggesting a concerted effort to advance treatment options as a central theme in recent years. Notably, since 2017, the keywords “continuous glucose monitoring,” “weight,” “progression,” “young adults,” “classification,” “time in range” has been more prominently concentrated, indicating that these issues currently represent the primary research focus and potentially mark a pivotal juncture with notable implications for future inquiry.

## 4. Discussion

### 4.1. General information

The findings of this study, derived from a comprehensive bibliometric analysis, underscore significant trends in blood glucose management for elderly diabetic patients. An observable increase in research output and a rise in international collaborations highlight a growing global interest in this area of health management. Institutional contributions have been substantial, with Harvard University and the University of Copenhagen emerging as prominent contributors. Harvard’s extensive research portfolio reflects a holistic approach to diabetes care, encompassing endocrinology, nutrition, and pharmaceutical development. In contrast, the University of Copenhagen prioritizes public health and clinical epidemiology, which are critical for comprehending the broader implications of diabetes management in older populations. The analysis indicates that journals such as *Diabetes Care*, *Diabetes*, and *Diabetologia* have published a considerable number of articles pertaining to blood glucose management for elderly diabetic patients. The preference of these journals for this research may be attributed to their targeted audience, editorial policies, and thematic alignment with current trends in diabetes care. The contributions of Beck Roy W are particularly noteworthy due to their significant scientific impact in this research domain, facilitating improved treatment outcomes and enhancing the quality of life for elderly diabetic patients. The time-overlay visualization map reveals a diverse array of subjects represented by both cited and citing journals, indicating untapped potential for further exploration within this research field and highlighting a paradigm shift from a focus on disease per se to outcomes and management among adult patients.

### 4.2. Research hotspots

The core of an academic field can be elucidated through its keywords, and the visual analysis of these keywords elucidates prevailing research trends and trajectories. Keywords facilitate a rapid understanding of the distribution and evolution of hotspots in blood glucose management research for elderly diabetic patients. According to the burst analysis of keywords, we conclude that related research primarily concentrates on the following aspects.

#### 4.2.1. Continuous glucose monitoring and time in range

Traditional self-monitoring of blood glucose necessitates the use of finger pricks, which can be inconvenient for patients. In contrast, continuous glucose monitoring (CGM) is a noninvasive technique that enables continuous assessment of glucose concentrations in subcutaneous interstitial fluid through a glucose sensor. This method allows for the real-time recording of blood glucose trends and fluctuations. Furthermore, for adults with type 2 diabetes, the 12-month effects of CGM have been shown to surpass those of self-monitoring of blood glucose in enhancing glucose control and numerous important health parameters. The utilization of CGM is associated with a reduction in average glucose levels from 184.0 to 147.2 mg/dL. Additionally, there are significant declines in HbA1c, body mass index, triglycerides, blood pressure, total cholesterol, diabetes distress, and the 10-year predicted risk for atherosclerotic cardiovascular disease.

The time in range (TIR) parameter signifies the proportion of time that blood glucose levels remain within designated glycemic thresholds. TIR is well-established as being correlated with the risk of diabetic complications, particularly the robust association between TIR and the risk of microvascular complications. As a result, TIR is increasingly regarded as a preferred metric for evaluating susceptibility to diabetic complications. Finally, the role of CGM in managing blood glucose levels among elderly patients with diabetes necessitates further validation through high-quality studies to improve its clinical application in the future.

#### 4.2.2. Weight management

Obesity is recognized as one of the most prevalent chronic conditions globally. Between 2017 and 2018, it was estimated that 42.4% of adults in USA were classified as obese, significantly increasing their risk for a multitude of chronic diseases, particularly type 2 diabetes (T2D). Consequently, effective weight management emerges as a critical strategy for the prevention and management of chronic diseases associated with obesity. Research indicates that for individuals identified as overweight or obese and prediabetic, the risk of developing diabetes decreases by approximately 16% for every kilogram of weight lost. A cohort study demonstrated that individuals with obesity who attempted weight loss—regardless of the method employed—generally experienced less weight gain and a reduced risk of developing diabetes. In contrast, lean individuals who intentionally lost weight often experienced greater weight gain and faced an increased risk of diabetes. While these studies suggest that moderate weight loss can enhance outcomes for diabetes mellitus in younger and middle-aged adults, there is a notable lack of high-quality evidence substantiating a robust relationship between obesity and diabetes mellitus in older adults. A recent study by Zhou et al reported that the association between obesity and T2D diminishes with age, indicating that elderly individuals may derive fewer benefits from weight loss compared to their younger counterparts. Considering this diminished association and the rising prevalence of geriatric syndromes among the elderly, clinicians must judiciously evaluate the benefits and potential drawbacks of weight loss interventions for older patients with T2D. Importantly, a loss of 5% or more of baseline weight has been independently associated with poorer performance on cognitive tests and an elevated risk of cardiovascular events in older adults. This finding highlights the necessity for elderly patients to pursue weight loss with caution and under the supervision of healthcare professionals.

### 4.3. Strengths and limitations

The strengths of the study reside in its comprehensive literature review, which utilized a reputable database and stringent inclusion criteria to ensure a relevant and focused analysis. By employing bibliometric techniques, the study offers an in-depth perspective on the research landscape, identifying key trends and influential publications. The analysis of keywords, particularly through burst detection, underscores thematic shifts and evolving research priorities over time. Nevertheless, the study is constrained by its reliance on a single database, which may overlook significant research outside its indexed scope or in non-English languages. Furthermore, the interpretation of keywords and citations, shaped by authors’ choices and indexing practices, may not consistently reflect the true essence of the content. Additionally, while the study’s findings are current, they may quickly become outdated in the rapidly advancing scientific landscape, necessitating ongoing analysis to capture emerging trends.

## 5. Conclusion

This study employs a comprehensive bibliometric analysis to evaluate the global research landscape, examine scholarly communication patterns, and identify key research hotspots in blood glucose management for elderly patients with diabetes. The primary areas of focus include noninvasive monitoring, personalized treatment, and geriatric-specific care models. This research provides an extensive overview of the current state of the field and offers valuable insights for researchers interested in diabetes management. Furthermore, it serves as a guide for future studies, highlighting the significance of exploring innovative approaches to care for the elderly diabetic population.

## Author contributions

**Data curation:** Li Zhou, Lubi He.

**Formal analysis:** Li Zhou, Lubi He.

## Supplementary Material


