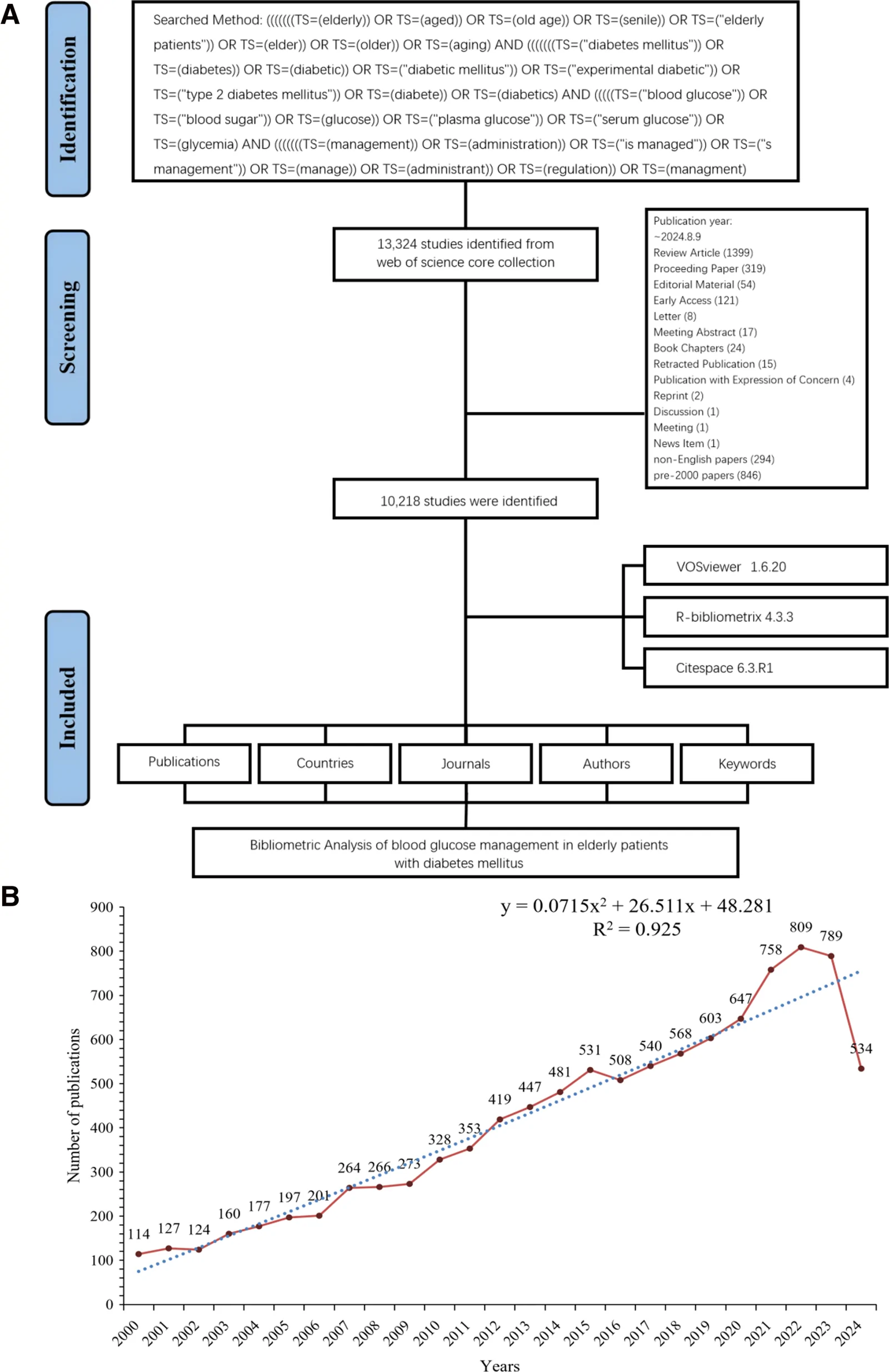# Global trends and hotspots in blood glucose management for elderly patients with diabetes. A bibliometric analysis (2000–2024): Erratum

**DOI:** 10.1097/MD.0000000000050753

**Published:** 2026-09-11

**Authors:** Li Zhou, Lubi He

In the article “Global trends and hotspots in blood glucose management for elderly patients with diabetes. A bibliometric analysis (2000–2024)”,^[1]^ which appears in Volume 104, Issue 33 of *Medicine*, Figure 1 was published with an incorrect number of articles (11,826). The number of articles has been corrected from 11,826 to 10,218 and the revised Figure 1 has now been updated in the online version.

**Figure:**