# Supplementary material for: Global trends and hotspots in blood glucose management for elderly patients with diabetes: A bibliometric analysis (2000–2024)
Source: Medicine (Baltimore). 2025 Aug 15;104(33):e43919. doi: 10.1097/MD.0000000000043919 (PMC12367009; doi:10.1097/MD.0000000000043919)
Supplement: Supplementary file 1 [file medi-104-e43919-s001.docx]

**Table S1** Publication and Citation Profiles of Leading Countries

| **Country** | **Articles** | **Freq** | **SCP** | **MCP** | **MCP_Ratio** | **TP** | **TP_rank** | **TC** | **TC_rank** | **Average Citations** |
| --- | --- | --- | --- | --- | --- | --- | --- | --- | --- | --- |
| **USA** | 2463 | 0.241 | 2047 | 416 | 16.90 | 10249 | 1 | 129377 | 1 | 52.5 |
| **China** | 1457 | 0.143 | 1215 | 242 | 16.60 | 5860 | 2 | 25439 | 3 | 17.5 |
| **Japan** | 633 | 0.062 | 568 | 65 | 10.30 | 2274 | 3 | 17617 | 4 | 27.8 |
| **United Kingdom** | 513 | 0.05 | 313 | 200 | 39.00 | 2227 | 4 | 26620 | 2 | 51.9 |
| **Australia** | 426 | 0.042 | 274 | 152 | 35.70 | 2053 | 5 | 15379 | 5 | 36.1 |
| **Italy** | 358 | 0.035 | 289 | 69 | 19.30 | 1647 | 6 | 11922 | 7 | 33.3 |
| **Canada** | 325 | 0.032 | 218 | 107 | 32.90 | 1398 | 8 | 9746 | 8 | 30 |
| **South Korea** | 318 | 0.031 | 276 | 42 | 13.20 | 1220 | 10 | 6770 | 11 | 21.3 |
| **Germany** | 296 | 0.029 | 181 | 115 | 38.90 | 1438 | 7 | 12102 | 6 | 40.9 |
| **Spain** | 238 | 0.023 | 197 | 41 | 17.20 | 1377 | 9 | 7356 | 10 | 30.9 |
| **India** | 230 | 0.023 | 193 | 37 | 16.10 | 737 | 14 | 5227 | 14 | 22.7 |
| **France** | 181 | 0.018 | 125 | 56 | 30.90 | 1092 | 11 | 5658 | 13 | 31.3 |
| **Turkey** | 165 | 0.016 | 157 | 8 | 4.80 | 539 | 19 | 2918 | 18 | 17.7 |
| **Denmark** | 158 | 0.015 | 95 | 63 | 39.90 | 814 | 12 | 5972 | 12 | 37.8 |
| **Brazil** | 154 | 0.015 | 117 | 37 | 24.00 | 621 | 18 | 2346 | 20 | 15.2 |
| **Iran** | 152 | 0.015 | 120 | 32 | 21.10 | 712 | 16 | 3307 | 17 | 21.8 |
| **Sweden** | 151 | 0.015 | 83 | 68 | 45.00 | 718 | 15 | 7791 | 9 | 51.6 |
| **Netherlands** | 141 | 0.014 | 96 | 45 | 31.90 | 738 | 13 | 5160 | 15 | 36.6 |
| **Poland** | 111 | 0.011 | 90 | 21 | 18.90 | 411 | 21 | 1393 | 26 | 12.5 |
| **Israel** | 101 | 0.01 | 75 | 26 | 25.70 | 421 | 20 | 2451 | 19 | 24.3 |

Note: Articles, Publications of Corresponding Authors only; Freq: Frequence of Total Publications; SCP: Single Country Publications; MCP: Multiple Country Publications; MCP_Ratio: Proportion of Multiple Country Publications; TP: Total Publications; TP_rank: Rank of Total Publications; TC: Total Citations; TC_rank: Rank of Total Citations; Average Citations, The average number of citations per publication.

**Table S2** Bibliometric Indicators of High-Impact Journals.

| **Journal** | **H_index** | **IF_2024** | **JCR_Quartile** | **PY_start** | **TP** | **TP_rank** | **TC** | **TC_rank** |
| --- | --- | --- | --- | --- | --- | --- | --- | --- |
| **Diabetes Care** | 99 | 14.8 | Q1 | 2000 | 308 | 1 | 30397 | 1 |
| **Diabetes** | 61 | 6.2 | Q1 | 2000 | 113 | 11 | 10766 | 2 |
| **Diabetologia** | 61 | 8.4 | Q1 | 2000 | 175 | 6 | 8716 | 4 |
| **Journal of Clinical Endocrinology & Metabolism** | 52 | 5 | Q1 | 2000 | 152 | 8 | 6486 | 5 |
| **Plos One** | 48 | 2.9 | Q1 | 2008 | 258 | 2 | 4007 | 11 |
| **Diabetes Research and Clinical Practice** | 40 | 6.1 | Q1 | 2000 | 236 | 3 | 4608 | 9 |
| **Diabetes Obesity & Metabolism** | 38 | 5.4 | Q1 | 2000 | 152 | 7 | 2842 | 14 |
| **Diabetic Medicine** | 37 | 3.2 | Q2 | 2000 | 199 | 4 | 5755 | 7 |
| **Diabetes Technology & Therapeutics** | 35 | 5.7 | Q1 | 2007 | 176 | 5 | 2772 | 15 |
| **Lancet Diabetes & Endocrinology** | 31 | 44 | Q1 | 2013 | 40 | 42 | 1621 | 26 |
| **Pediatric Diabetes** | 30 | 3.9 | Q1 | 2004 | 119 | 10 | 2220 | 19 |
| **Cardiovascular Diabetology** | 28 | 8.5 | Q1 | 2003 | 74 | 18 | 1154 | 39 |
| **Metabolism-Clinical and Experimental** | 28 | 10.8 | Q1 | 2000 | 68 | 21 | 1988 | 22 |
| **Clinical Therapeutics** | 27 | 3.2 | Q2 | 2000 | 59 | 27 | #N/A | #N/A |
| **Endocrinology** | 26 | 3.8 | Q2 | 2000 | 42 | 40 | 1663 | 25 |
| **Lancet** | 26 | 98.4 | Q1 | 2000 | 27 | 58 | 6325 | 6 |
| **Diabetes Educator** | 25 | #N/A | #N/A | 2000 | 59 | 28 | #N/A | #N/A |
| **Experimental and Clinical Endocrinology & Diabetes** | 24 | 1.6 | Q4 | 2000 | 66 | 23 | 482 | 107 |
| **BMC Public Health** | 22 | 3.5 | Q1 | 2007 | 66 | 22 | 878 | 55 |
| **Diabetes & Metabolism** | 22 | 4.6 | Q1 | 2000 | 68 | 20 | 1224 | 36 |

Note(s): H_index: The h-index of the journal, which measures both the productivity and citation impact of the publications. IF 2024: Impact Factor, indicating the average number of citations to recent articles published in the journal. JCR_Quartile: The quartile ranking of the journal in the Journal Citation Reports, indicating the journal's ranking relative to others in the same field (Q1: top 25%, Q2: 25%-50%, Q3: 50%-75%, Q4: bottom 25%). TP: Total Publications. TP_rank: Rank of Total Publications. TC: Total Citations. TC_rank: Rank of Total Citations. Average Citations: The average number of citations per publication. PY_start: Publication Year Start, indicating the year the journal started publication.

**Table S3** Publication and Citation Profiles of High-Impact Authors.

| **Authors** | **H_index** | **g-index** | **m-index** | **PY_start** | **TP** | **TP_Frac** | **TP_rank** | **TC** | **TC_rank** |
| --- | --- | --- | --- | --- | --- | --- | --- | --- | --- |
| **Beck Roy W** | 22 | 30 | 1.29 | 2008 | 30 | 3.91 | 2 | 6253 | 1 |
| **Khunti Kamlesh** | 22 | 42 | 1.29 | 2008 | 47 | 5.63 | 1 | 1812 | 11 |
| **Danne Thomas** | 16 | 19 | 1.33 | 2013 | 19 | 1.70 | 19 | 1196 | 15 |
| **Davies Melanie J** | 16 | 27 | 1.00 | 2009 | 27 | 3.31 | 3 | 1032 | 18 |
| **Hood Korey K** | 15 | 26 | 1.00 | 2010 | 26 | 4.46 | 4 | 1215 | 14 |
| **Kollman Craig** | 15 | 19 | 0.88 | 2008 | 19 | 1.55 | 23 | 3254 | 3 |
| **Bi Yufang** | 14 | 24 | 0.93 | 2010 | 24 | 1.65 | 7 | 715 | 28 |
| **Davies M J** | 14 | 22 | 0.88 | 2009 | 22 | 3.12 | 9 | 846 | 25 |
| **Jia Weiping** | 14 | 20 | 0.88 | 2009 | 20 | 1.88 | 16 | 724 | 27 |
| **Khunti K** | 14 | 22 | 0.93 | 2010 | 22 | 2.82 | 10 | 708 | 29 |
| **Ning Guang** | 14 | 26 | 0.93 | 2010 | 26 | 1.72 | 6 | 966 | 21 |
| **Wang Weiqing** | 14 | 22 | 1.00 | 2011 | 22 | 1.42 | 11 | 947 | 23 |
| **Holst Jens J** | 13 | 21 | 0.72 | 2007 | 21 | 2.46 | 12 | 1231 | 13 |
| **Hovorka Roman** | 13 | 19 | 0.77 | 2008 | 19 | 2.39 | 21 | 548 | 38 |
| **Maahs David M** | 13 | 23 | 1.00 | 2012 | 23 | 2.89 | 8 | 2791 | 6 |
| **Ryden Lars** | 13 | 14 | 0.72 | 2007 | 14 | #REF! | 50 | 1134 | 17 |
| **Berg Cynthia A** | 12 | 19 | 0.86 | 2011 | 19 | 3.25 | 18 | 427 | 47 |
| **Eriksson Johan G** | 12 | 19 | 0.63 | 2006 | 19 | 2.40 | 20 | 571 | 37 |
| **Hirsch Irl B.** | 12 | 13 | 0.63 | 2006 | 13 | #N/A | #N/A | 2858 | 5 |
| **Li Tsai-Chung** | 12 | 19 | 0.92 | 2012 | 19 | 2.38 | 24 | 513 | 40 |

Note(s): H_index: The h-index of the journal, which measures both the productivity and citation impact of the publications. g_index: The g-index of the journal, which gives more weight to highly-cited articles. m_index: The m-index of the journal, which is the h-index divided by the number of years since the first published paper. TP: Total Publications. TP_rank: Rank of Total Publications. TP_Frac: Fraction of Total Publications TC: Total Citations. TC_rank: Rank of Total Citations. PY_start: Publication Year Start, indicating the year the journal started publication.

**Table S4. Top 20 cited papers.**

| **Paper** | **DOI** | **Total Citations** | **TC per Year** | **Normalized TC** |
| --- | --- | --- | --- | --- |
| Knowler Wc, 2002, New Engl J Med | 10.1056/nejmoa012512 | 4632 | 201.39 | 39.36 |
| Danaei G, 2011, Lancet | 10.1016/S0140-6736(11)60679-X | 2651 | 189.36 | 49.14 |
| Toobert Dj, 2000, Diabetes Care | 10.2337/diacare.23.7.943 | 1679 | 67.16 | 18.15 |
| Schauer Pr, 2012, New Engl J Med | 10.1056/NEJMoa1200225 | 1606 | 123.54 | 32.69 |
| Landon Mb, 2009, New Engl J Med | 10.1056/NEJMoa0902430 | 1491 | 93.19 | 29.16 |
| Bhatt Dl, 2006, Jama-J Am Med Assoc | 10.1001/jama.295.2.180 | 1323 | 69.63 | 23.86 |
| Schauer Pr, 2014, New Engl J Med | 10.1056/NEJMoa1401329 | 1192 | 108.36 | 30.29 |
| Zampetaki A, 2010, Circ Res | 10.1161/CIRCRESAHA.110.226357 | 1169 | 77.93 | 22.09 |
| Foster Nc, 2019, Diabetes Technol The | 10.1089/dia.2018.0384 | 1138 | 189.67 | 42.55 |
| Tamborlane Wv, 2008, New Engl J Med | NA | 1066 | 62.71 | 17.81 |
| Saydah Sh, 2004, Jama-J Am Med Assoc | 10.1001/jama.291.3.335 | 1015 | 48.33 | 10.97 |
| Michael Md, 2000, Mol Cell | 10.1016/S1097-2765(00)00010-1 | 983 | 39.32 | 10.63 |
| Miller Km, 2015, Diabetes Care | 10.2337/dc15-0078 | 938 | 93.8 | 26.84 |
| Musso G, 2011, Ann Med | 10.3109/07853890.2010.518623 | 926 | 66.14 | 17.17 |
| Mcintyre Hd, 2019, Nat Rev Dis Primers | 10.1038/s41572-019-0098-8 | 893 | 148.83 | 33.39 |
| Nathan Dm, 2008, Diabetes Care | 10.2337/dc08-0545 | 841 | 49.47 | 14.05 |
| Rawshani A, 2018, New Engl J Med | 10.1056/NEJMoa1800256 | 839 | 119.86 | 32.93 |
| Tuttle Kr, 2014, Am J Kidney Dis | 10.1053/j.ajkd.2014.08.001 | 818 | 74.36 | 20.78 |
| HU G, 2004, ARCH INTERN MED-A | 10.1001/archinte.164.10.1066 | 798 | 38 | 8.63 |
| Azziz R, 2016, Nat Rev Dis Primers | 10.1038/nrdp.2016.57 | 780 | 86.67 | 23.04 |
